# Cross-cultural adaptation and validation of the Chinese version of the modified Fresno test for physical therapists

**DOI:** 10.1186/s12909-024-06615-4

**Published:** 2025-01-02

**Authors:** Li-Yang Chen, Yi-Jing Lue, Po-Hsun Wu, Yi-Liang Kuo

**Affiliations:** 1 Department of Rehabilitation, Dalin Tzu Chi Hospital, Buddhist Tzu Chi Medical Foundation, Chaiyi, 62247 Taiwan; 2 Department of Physical Therapy, Kaohsiung Medical University, Kaohsiung City, 807 Taiwan; 3 Department of Medical Research, Kaohsiung Medical University Hospital, Kaohsiung City, 807 Taiwan; 4 Department of Rehabilitation, Yonghe Cardinal Tien Hospital, New Taipei City, 234 Taiwan; 5 Department of Physical Therapy, College of Medicine, National Cheng Kung University, Tainan City, 701 Taiwan

**Keywords:** Evidence-based practice, Physical therapy, Fresno test, Reproducibility of results

## Abstract

**Background:**

The modified Fresno test was developed to assess knowledge and skills related to evidence-based practice among physical therapists and students, however no Chinese version is available. Therefore, the aim of this study was to cross-culturally adapt the English version of the modified Fresno test into Chinese and to evaluate its validity and reliability.

**Methods:**

This was a cross-sectional validation study. During Phase 1 cross-cultural adaptation, forward translation, synthesis, back-translation, expert review, and prefinal test were carried out. Content validity was analyzed. During Phase 2 validation, two groups representing different levels of evidence-based practice competence completed the tests twice, two weeks apart. Known-group validity, internal consistency, and test-retest reliability were analyzed.

**Results:**

Phase 1: According to the expert review, the Chinese version of the modified Fresno test demonstrated good content validity (item-level content validity index = 0.93 to 1.0; scale-level content validity index = 0.99). Phase 2: The expert group scored (115.3 ± 36.2) significantly higher than the novice group did (79.7 ± 25.8) for the entire test (*p* = 0.01), suggesting good known-group validity. However, the superior performance of the expert group over the novice group was not consistently found across all the questions on the test. In addition, the internal consistency was good (Cronbach’s α = 0.96) and the test-retest reliability (ICC_2,1_ = 0.89, 95% CI = 0.81 to 0.94) was good to excellent.

**Conclusions:**

The Chinese version of the modified Fresno test is a valid and reliable tool for objectively assessing evidence-based practice knowledge and skills in Chinese-speaking physical therapists and students.

**Supplementary Information:**

The online version contains supplementary material available at 10.1186/s12909-024-06615-4.

## Introduction

Over the years, evidence-based practice (EBP) has become a crucial foundation for optimal patient care [1]. EBP involves integrating the best research evidence, clinical expertise, and patient preferences to make clinical decisions [2]. By implementing EBP, healthcare professionals can provide more effective and safer treatment to patients, reducing the waste of healthcare resources and maintaining socioeconomic benefits [3]. However, many factors can influence implementation of EBP, such as lack of knowledge and skills [4], time constraints [5], and burnout [6].

There are several assessment tools available to assess EBP competence given the importance of teaching and translating knowledge and skills into clinical practice. Shaneyfelt et al. identified 104 instruments evaluating knowledge, skills, attitude, and/or other domains of EBP in their systematic review [7]. Later, Kumaravel et al. reported and categorized 12 new instruments according to the EBP steps and educational outcomes evaluated in an updated systematic review [8]. Among all available instruments, the Fresno test is the most commonly used. The Fresno test was developed and validated by Ramos et al. as a valid and reliable test for knowledge and skills related to EBP among physicians [9]. The Fresno test uses open-ended questions to assess the first four steps of EBP including asking a question, acquiring evidence, critically appraising evidence, and applying evidence in the context of realistic scenarios. The answers are scored via the standardized grading rubrics. The open-ended questions and standardized grading enable the Fresno test to objectively measure the knowledge and skills of EBP.

As EBP evolves across various professions, the Fresno test has been adapted to different versions applicable to other healthcare fields, such as physical therapy [10–12], nursing [13–15], speech therapy [16, 17], and occupational therapy [18], as well as different languages, such as Chinese [19], Spanish [20], Turkish [21], and Portuguese [15]. The modified Fresno test (MFT) is specifically modified by Tilson et al. in 2010 for evaluating the knowledge and skills of EBP among physical therapists and students [10]. In addition to the physical therapy-specific modification of the clinical scenarios, the question stems, the scoring rubric, and two new short answer questions were added. In the validation study of Tilson et al., the MFT demonstrated acceptable internal consistency (Cronbach’s α = 0.78), excellent interrater reliability (ICC_2,1_ = 0.91), and excellent intrarater reliability (Rater 1: ICC_2,1_ = 0.95, Rater 2: ICC_2,1_ = 0.96) [10].

Validity and reliability are two fundamental properties in any measurement instrument. Although the original version of the MFT has shown satisfactory validity and reliability results in the English-speaking population [10], the test is not necessarily suitable for use in the Chinese-speaking population. The MFT has been translated into the Portuguese language [11], but no Chinese version is available. Therefore, the purpose of this study was to cross-culturally adapt and validate the Chinese version of the MFT.

## Methods

The study comprised two phases: cross-cultural adaptation (phase 1) and validation (phase 2). The research protocol underwent ethical review by the Institutional Review Board of National Chung Cheng University with approval under protocol number CCUREC111090501. All participants provided informed consent to participate in this study.

### Modified Fresno test

The MFT comprises two clinical scenarios, eight short-answer questions, two calculation questions, and three fill-in-the-blank questions [10]. The participants must first select a clinical scenario and then answer seven questions related to the chosen clinical scenario, including formulate question (Q1), information sources (Q2), best study design for intervention (Q3), search (Q4), relevance (Q5), internal validity (Q6), and effect size (Q7). Following these questions, the test includes questions on the patient/family preferences (Q8), sensitivity (Q9), absolute risk reduction (Q10), confidence intervals (Q11), best research design for diagnosis (Q12), and best research design for prognosis (Q13). The participants were required not to consult any external resources during the one-hour tests or during the two-week period between the first and second tests.

Each question of the MFT is scored via the standardized scoring rubrics [10]. The answers are rated as excellent, strong, limited, minimal, or not evident based on the participant’s response. Seven questions (Q1-7) out of the 13 questions on the MFT are scored from 0 to 24, two questions (Q8 and Q10) are scored from 0 to 16, one question (Q9) is scored from 0 to 12, and three questions (Q11-13) are scored 0 or 4. The maximum total score is 224 points, with higher scores indicating better knowledge and skills of EBP.

### Phase 1: cross-cultural adaptation

The translation procedure adhered to the guidelines recommended by Beaton and Bombardier [22]. After approval was obtained from Dr. Tilson, the original author of the English version of the MFT [10], the following steps were conducted.

Step 1 − Forward translation: The MFT was translated from English to Chinese by two translators independently. Translator one whose first language was Chinese had completed graduate studies and had worked in English-speaking countries for 8 years. The translator also had EBP teaching experience in higher education. Translator two, whose native language is Chinese, was a professional from Wallace Academic Editing Company and was not aware of the study’s purpose, the test concepts, or EBP knowledge.

Step 2 − Synthesis of translation: Two forward translation versions (FT_1_, FT_2_) were compared and synthesized into a single translation (FT_12_) by a senior researcher who had background in physical therapy and was knowledgeable in EBP.

Step 3 − Expert review: Five EBP experts from various fields including medicine, nursing, physical therapy, and library information independently evaluated the original English version and the synthesis of forward translation (FT_12_). All EBP experts were asked to identify any semantic or content inequivalence in the translation and provide suggestions. The agreed translation was obtained through group discussions and consensus in online meetings.

Step 4 − Backward translation: Two native English speakers who were also proficient in Chinese but lacking a medical background or any knowledge of EBP concepts independently translated the FT_12_ version from Chinese to English (BT_1_, BT_2_). Two backward translations were synthesized into a single translation (BT_12_) by the same senior researcher as in Step 2. The research team summarized the inequivalences between the original English version, the forward (FT_12_) translation, and the backward (BT_12_) translation. This information, along with all versions of the MFT, was then mailed to the EBP experts for further review. The prefinal version of the Chinese MFT was finalized through group discussions and consensus in online meetings.

Step 5 − Prefinal test: A convenience sample of 15 physical therapy graduates and 15 physical therapy students (Table 1) was recruited to test the prefinal version of the Chinese MFT online. The participants were also asked to evaluate the comprehensibility of the translated MFT and provide written feedback and suggestions. The inclusion criterion for physical therapy graduates was graduation from any physical therapy program at a junior college or university in Taiwan, without limitations based on age, years since graduation, or possession of a physical therapist license. The inclusion criterion for physical therapy students was the completion of mandatory EBP courses at school, without age or academic year restrictions. The research team summarized participants’ feedback and suggestions and then discussed with the EBP experts whether and how to modify the translation to strengthen its meaning. The Chinese version of the MFT (Supplementary) was finalized through group discussion and consensus.

**Table 1:** Demographic data of participants

Characteristics	Phase			
	Phase 1	Phase 2		
	Pre-final test	Internal consistency	Known-group validity	
	Total (*n* = 30)	Total (*n* = 46)	Expert (*n* = 13)	Novice (*n* = 33)
Gender (Male, Female)	13, 17	14, 32	2, 11	12, 21
Age (years)	23.4 ± 2.7	27.8 ± 10.4	41.9 ± 9.5	21.9 ± 2.4
Occupation				
School faculty	0	6	6	0
Clinical staffs	15	7	7	0
Students	15	33	0	33
Highest education				
Doctorate	1	6	6	0
Master	6	4	4	0
Bachelor	23	36	3	0
Undergraduate	0	0	0	33^a^
Working experience in physical therapy (year)				
0	15	33	0	33
1–10	15	4	4	0
≥ 11	0	9	9	0
Credits earned from undergraduate EBP courses				
< 1	13	18	10	8
1–2	10	26	1	25
> 2	7	2	2	0
Teaching experience in EBP courses (years)				
0	30	33	0	33
1–5	0	9	9	0
≥ 6	0	4	4	0
Hours of EBP continuing education				
0	25	39	8	31
1–5	4	4	2	2
≥ 6	1	3	3	0

^a^ Number includes undergraduate students

### Phase 2: validation

**Fig. 1:**
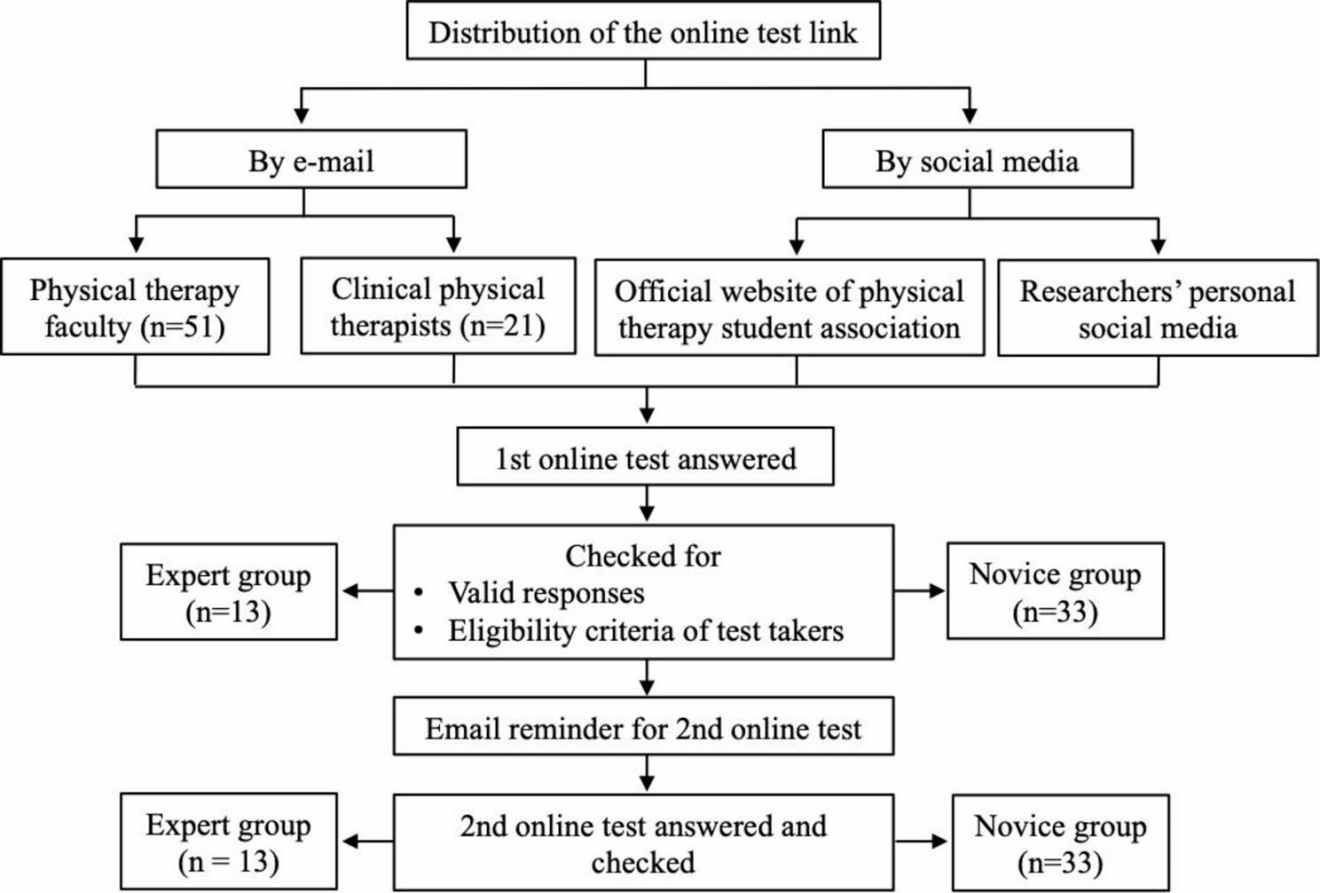
Flow diagram for phase 2 of the study

To establish the validity and reliability of the Chinse version of the MFT, two groups of participants (an expert group and a novice group) were recruited via the convenience sampling (Fig. 1). The inclusion criterion for the expert group was faculty members in physical therapy or clinical physical therapists with more than one year of EBP teaching experience; the inclusion criterion for the novice group was physical therapy students who had completed compulsory EBP courses at school. The required sample size was determined on the basis of a respondent-to-item ratio of 5:1 [23]. Thus, to validate the Chinese version of the MFT, which contains a total of 13 items, a minimum of 66 participants were needed. All participants provided consent to participate in the study.

The MFT test was administered twice online, with two weeks apart. The participants were informed of the test link through social media platforms or emails and were required to complete the test within one hour. Two weeks after the initial test, the participants were reminded via email to complete the Chinese version of the MFT for the second time.

Before scoring the Fresno test, two junior researchers (LYC and PHW) received training from the senior researcher (YLK). Both junior researchers carefully reviewed the scoring rubrics [10] and independently scored five samples. The score results were compared and discussed with a senior researcher. After training, both junior researchers scored the tests independently. If there was any disagreement between the score results, a decision was made by discussion or consultation with the senior researcher.

### Validity and reliability

The validity of the MFT was evaluated via an assessment of content validity and construct validity (known-group validity). During Phase 1, the experts assessed content validity for each question of the MFT based on three criteria (content suitability, translation accuracy, and text clarity). The experts’ responses were recorded on a four-point Likert scale, categorized as follows: 4 indicated “very appropriate, no further modifications needed”; 3 indicated “appropriate, some modifications should be made”; 2 indicated “inappropriate, significant modifications required”; and 1 indicated “highly inappropriate, requiring retranslation”. The scores assigned to each question were used to calculate the content validity index (CVI) [24]. During Phase 2, known-group validity was established by evaluating the ability of the Chinese version of the MFT to discriminate between the expert and novice groups with different levels of EBP competence. The reliability of the Chinese version of the MFT was evaluated by assessing its internal consistency and test-retest reliability.

### Statistical analysis

Descriptive statistics were used to summarize demographic characteristics and scores on items of the test.

To assess the content validity, the item-level and scale-level CVIs [25] were calculated. The item-level CVI represents the content validity for items of the test, and the scale-level CVI refers to the content validity for the entire test. To assess known-group validity, independent t-tests were used to compare the scores of the first test between the expert and novice groups [26]. The level of statistical significance was set at *p* < 0.05.

Internal consistency was analyzed via Cronbach’s α coefficient, with values greater than 0.7 considered acceptable [27]. Test-retest reliability for the entire test was analyzed via the intraclass correlation coefficient (ICC_2,1_) [28]. Test-retest reliability for items of the test was analyzed via the weighted Cohen’s kappa for ordinal ratings and unweighted Cohen’s kappa for nominal ratings [29].

Data entry and statistical analyses were performed via the SPSS software version 20.0 for Windows (SPSS Inc., Chicago, IL, USA).

## Results

### Phase 1

#### Content validity

Most items in the MFT were translated into Chinese without difficulty. All experts rated the instructions, two scenarios, and all the questions of the translation as appropriate or very appropriate in terms of content suitability, translation accuracy, and text clarity of the test, except for Question 9. The item-level CVI for the questions 1–8 and 10–13 was a perfect 1. For question 9, which asks for statistics about diagnosis (sensitivity, positive predictive value, and positive likelihood ratio), one expert expressed concerns about its applicability to physical therapists and students and rated the content suitability as highly inappropriate. Despite this rating, the item-level CVI for question 9 was 0.93, indicating substantial agreement among all the experts. The scale-level CVI for the entire test was notably high at 0.99.

In the prefinal test, 11 out of 30 participants provided a total of 23 suggestions. Three participants reported difficulties answering question 11 because they did not know how many details should be included when giving an example of a confidence interval that indicates statistical significance. The research team consulted with the experts and modified the translation to improve comprehension. The other 20 suggestions are requests for providing answer options, requests for information not included in the original test (e.g., provide the age for the case in Clinical Scenario 1 and the search fields for Question 4), and unclear or unspecific suggestions (e.g., comment that the text description should be more precise and concise without specifying which part of the test). The first two types of suggestions were beyond cross-cultural adaptation, while the last type was too vague. Therefore, these suggestions were not adopted to modify the translation.

### Phase 2

Forty-six participants were recruited for the validation phase of this study, with 13 in the expert group and 33 in the novice group (Table 1). All the participants completed both the initial test (mean score = 89.8 ± 33.2) and the follow-up test (mean score = 91.3 ± 33.0). On average, the expert and novice groups took 39.2 (range 17–59) and 30.4 (range 12–58) minutes to complete the test, respectively.

#### Known-group validity

The mean score of the entire test for the expert group (115.3 ± 36.2) was significantly higher than the mean score for the novice group (79.7 ± 25.8) (*t* = 3.72, *p* = 0.001). The mean difference between the two groups was 35.6 points (95% CI = 16.3 to 54.9). Table 2 lists the results of the independent *t* test for individual questions of the test. Among the total 13 questions, the expert group outperformed the novice group in the questions 3 (best study design for intervention), 4 (search), 6 (internal validity), 8 (patient/family preferences), 9 (sensitivity), and 10 (absolute risk reduction) (*p* < 0.05). There were no significant between-group differences in the other seven questions.

**Table 2:** Results of independent *t*-test for individual questions of the test between the expert and novice groups

Question	Expert group	Novice group	Mean Difference (95% CI)	t	*p*
1	14.3 ± 7.0	11.6 ± 6.4	2.7 (-1.6 to 7.1)	1.28	0.21
2	13.1 ± 7.7	10.2 ± 6.3	2.9 (-1.5 to 7.3)	1.32	0.19
3	14.8 ± 3.6	10.8 ± 5.8	4.0 (0.5 to 7.4)	2.28	0.03
4	14.0 ± 5.3	8.8 ± 5.2	5.2 (1.8 to 8.7)	3.07	0.01
5	11.7 ± 7.9	8.6 ± 5.5	3.1 (-1.1 to 7.2)	1.50	0.14
6	10.0 ± 7.2	5.4 ± 5.4	4.6 (0.7 to 8.5)	2.36	0.02
7	6.1 ± 6.5	6.4 ± 4.4	-0.3 (-3.6 to 3.1)	0.17	0.86
8	7.1 ± 3.6	3.9 ± 1.5	3.1 (0.9 to 5.4)	4.17	0.01
9	7.6 ± 4.1	4.4 ± 4.3	3.3 (0.5 to 6.0)	2.35	0.02
10	10.9 ± 5.2	5.0 ± 4.7	5.8 (2.6 to 9.0)	3.67	0.01
11	0.9 ± 1.8	0.2 ± 1.0	0.7 (-0.4 to 1.8)	1.69	0.21
12	2.2 ± 2.1	1.9 ± 2.0	0.2 (-1.1 to 1.6)	0.32	0.75
13	2.8 ± 1.9	2.4 ± 2.0	0.4 (-1.0 to 1.6)	0.54	0.60
Total	115.3 ± 36.2	79.7 ± 26.2	35.6 (16.3 to 54.9)	3.72	0.01

Note: formulate question (Q1), information sources (Q2), best study design for intervention (Q3), search (Q4), relevance (Q5), internal validity (Q6), and effect size (Q7). Following these questions, the test includes questions on the patient/family’s preference (Q8), sensitivity (Q9), absolute risk reduction (Q10), confidence interval (Q11), best research design for diagnosis (Q12), and best research design for prognosis (Q13)

#### Reliability

The Cronbach’s α for the whole sample in the first test was 0.96. The test-retest reliability of the total test score was good to excellent (ICC_2,1_ = 0.89, 95% CI = 0.81 to 0.94). The average kappa for the 13 questions was 0.6 (range 0.1–0.9). Table 3 lists the results of Cronbach’s α if individual test item were deleted, along with the kappa statistics for each individual question.

**Table 3:** Internal consistency and test-retest reliability of individual questions of the test

Question	Cronbach’s α if item deletes	Cohen’s kappa
1. Formulate question		
a: Population	0.96	0.58
b: Intervention	0.96	0.92
c: Comparison	0.95	0.76
d: Outcome	0.95	0.88
2. Information sources		
a: Variety of sources	0.96	0.65
b: Convenience	0.95	0.46
c: Clinical relevance	0.95	0.12
d: Validity	0.95	0.75
3. Best design for intervention		
a: Study design	0.96	0.51
b: Justification	0.96	0.34
4. Search		
a: Search terms	0.96	0.82
b: Tags/Strategy	0.96	0.45
c: Delimiters	0.95	0.65
5. Relevance		
a: The question	0.96	0.35
b: Description of subjects	0.96	0.63
6. Internal validity	0.96	0.58
7. Effect size		
a: Magnitude	0.95	0.68
b: Statistical significance	0.96	0.61
8. Patient/family’s preference		
a: Question 1	0.96	0.43
b: Question 2	0.96	0.15
9. Sensitivity		
a: Sensitivity	0.95	0.77
b: Positive predictive value	0.95	0.70
c: Positive likelihood ratio	0.95	0.38
10. Absolute risk reduction		
a: Absolute risk reduction	0.95	0.63
b: Relative risk reduction	0.95	0.67
c: Number needed to treat	0.95	0.73
d: p value	0.96	0.51
11. Confidence interval	0.95	0.90
12. Best research design for diagnosis	0.95	0.57
13. Best research design for prognosis	0.95	0.40

## Discussion

The results of this study demonstrate that the Chinese version of the MFT is a valid and reliable tool for objectively assessing the knowledge and skills of EBP among physical therapists and students. Although many instruments are available to assess EBP competence, most are self-reported questionnaires. For example, the Evidence-based Practice Questionnaire (EBPQ), developed by Upton and Upton [30], requires users to subjectively rate their frequency of practice EBP, attitude towards EBP, and knowledge and skill in EBP on a 7-point Likert scale. The self-reported instruments have the advantage of being quick and easy to administer; however, users are likely to under- or over-estimate their own competence. In contrast, objective instruments, such as the Fresno test [9] and its different versions [10–19] including this Chinese version of the MFT, contain open-ended questions to measure higher-order thinking and knowledge of EBP.

### Content validity

The results of the CVIs in this study suggest good content validity based on the cutoff values suggested by Polit et al. (item-level CVI > 0.78, scale-level CVI > 0.9) [31]. Tilson et al. [10] and Silva et al. [11] did not calculate the CVI in their validation studies for the MFT, posing difficulties for comparisons with previous research in the field of physical therapy. Conversely, Cakmakkaya et al. translated the FT into the Turkish language and reported excellent content validity with all item-level CVIs being 1 [21]. Halm et al. adapted the FT for emergency room nurses and reported the item-level CVIs ranging between 0.75 and 1 [14]. The content validity of the Chinese version of the MFT is similar to that reported in previous studies in the medical and nursing fields.

During the expert review, an EBP expert from the medical field expressed reservations about the appropriateness of question 9 for physical therapists and students. However, the consensus among the other four experts was to make no changes. Given the importance for physical therapists and students in understanding diagnostic accuracy [32, 33], the question 9 was consequently retained in the prefinal test.

### Known-groups validity

This study demonstrated that the Chinese version of the MFT effectively distinguished physical therapists and students with different levels of EBP competence, as evidenced by a significantly higher total test score in the expert group than in the novice group. Previous studies examining the known-group validity of the Fresno test [9] and MFT [10] reported similar results, indicating that the original Fresno test and its different versions can discriminate between individuals with different levels of EBP competence.

Although the expert group outperformed the novice group for the entire test and for some specific questions in this study, unexpected results were found when the total scores of this study and previous studies were closely compared. The novice group in this study who completed compulsory EBP courses at school had a significantly lower mean MFT score (79.7) than did the freshman students in the study of Miller et al. [34] (109) and the first-year doctoral students in the study of Tilson et al. [10] (92.8). Similarly, the expert group in this study (115.3) scored lower than the EBP expert did in the study of Tilson et al. [10] (149). In addition, the mean score of two (Q6 internal validity, Q8 patient/family preferences) of the six questions in which the expert group significantly outperformed the novice group was not greater than 50% of the available points for individual questions. This cutoff value was defined by Tilson et al. as a passing score [10]. This finding raises the question: Is the test difficulty of the MFT appropriate for physical therapists and students in Taiwan? Nevertheless, using the same measurement tool is crucial, as it allows for comparisons across different countries. From a professional development perspective, this finding suggests that physical therapy professionals in Taiwan, including experts, should invest more time and effort learning EBP knowledge and skills, as there is significant room for improvement in EBP competence. Furthermore, it may be crucial to evaluate individual question responses and scores to assess knowledge and skill related to EBP accurately among physical therapists.

The superior performance of the expert group over the novice group was not consistently found across all the questions on the test. Notably, the novice group’s score for question 7 asking effect size was slightly higher than that of the expert group although the difference was not statistically significant. Previous studies have similarly shown that certain questions in the Fresno test cannot be used to differentiate study groups with different levels of EBP competence [9, 10]. The rationale for retaining these questions was to deliberately avoid making the test overly challenging and to ensure a more comprehensive assessment of EBP competency.

### Internal consistency

Cronbach’s α measures the relationship between a group of questions within a scale. Compared with the original version of the Fresno test (Cronbach’s α = 0.88) [9] and Tilson’s English version of the MFT for physical therapy (Cronbach’s α = 0.78) [10], the Cronbach’s α of 0.96 in this study indicates that the internal consistency of the Chinese version of the MFT is excellent [27].

### Test-retest reliability

Based on the 95% CI of the ICC value [28], the Chinese version of the MFT demonstrated good to excellent test-retest reliability for the total test score. This result was better than a similar study by Miller et al. [34] which assessed the EBP abilities of first-year physical therapy students (ICC_2,1_= 0.46, 95% CI = 0.16 to 0.69). The low kappa values for some individual questions may be related to inconsistent performance of the participants. For example, when asking to compare the pro and cons of different information resources (questions 2c), some participants answered clinical relevance during the first test but did not mention clinical relevance during the follow-up test.

### Limitations

This study faced several limitations. First, this study initially aimed to recruit 33 participants for the expert group through an email invitation to 72 eligible experts, but only 13 experts were ultimately enrolled. Excessive work-related stress and burnout experienced by the eligible experts [6] might have influenced their willingness to participate in the study. Time constraints and concerns about not performing well may be other reasons. As a result, the total number of participants in this study did not meet the priori calculated sample size based on a response-to-item ratio of 5:1 or the COSMIN (COnsensus-based Standards for the selection of health Measurement INstruments) recommendation of at least 100 participants for cross-cultural validity [35]. This small sample size may have influenced the validation process of the Chinese version of the MFT, impacting the representativeness and generalizability of the findings. The small sample size, particularly in the expert group, may have affected the analysis of known-group validity through independent t-tests. It also prevented the conduct of confirmatory factor analysis to assess cross-cultural validity.

Additionally, the test was conducted online. While this method saves time and commuting costs for researchers, it does not guarantee that participants answer the questions sincerely. There is a possibility that participants might have referred to external sources or conducted online searches while answering the questions, which could have potentially compromised the accuracy of the test. Future studies should aim for a larger sample size and conduct in-person testing. These steps could significantly improve the understanding of the knowledge and skills of EBP among physical therapists and students.

## Conclusions

The Chinese version of the MFT has good content validity, good known-group validity, excellent internal-consistency, and good to excellent test-retest reliability. The Chinese version of the MFT is a valid and reliable tool for objectively assessing the knowledge and skills related to EBP among physical therapists and students in the Chinese-speaking population.

## Electronic supplementary material

Below is the link to the electronic supplementary material.

Supplementary Material 1

## Data Availability

The datasets used and/or analyzed during the current study are available from the corresponding author upon reasonable request.

## Abbreviations

EBP: Evidence-based practice
CVI: Content validity index
MFT: Modified Fresno test
ICC: Intraclass correlation coefficient
CI: Confidence interval
