# Supplementary material for: Cross-cultural adaptation and validation of the Chinese version of the modified Fresno test for physical therapists
Source: BMC Med Educ. 2025 Jan 2;25:1. doi: 10.1186/s12909-024-06615-4 (PMC11694388; doi:10.1186/s12909-024-06615-4)
Supplement: Supplementary file 1 — Supplementary Material 1 [file 12909_2024_6615_MOESM1_ESM.pdf]

中文版修正式夫勒斯諾量表

說明：

實證實踐(Evidence Based Practice，簡稱 EBP)包含與搜尋及評估證據以告知臨床實踐相關的知識與技能。本測驗旨在評估您的實證實踐能力。本測驗共有 8 題簡答題，2 題計算題，以及 3 題填空題。作答時，您僅能使用計算機與紙筆，不得使用其它資源(如網路、書籍等)。請在 60 分鐘內，一次完成整個測驗。

請根據以下臨床情境回答第 1 至 4 題與第 8 題：

情境 1：

您剛評估完小美的狀況。她是一位秘書，3 天前在工作時搬動 10 個 11 公斤重的文件箱，導致下背部損傷。她的 X 光片診斷結果無異常，症狀已有緩解，目前唯一的症狀是彎腰與久坐時，會引發下背疼痛，疼痛強度為 2/10。她已經請假休息兩天，雖然很想回去上班，但也擔心再次受傷。您正在考慮為她進行穩定運動訓練，但想知道是否應將徒手治療納入小美的物理治療計畫中。

情境 2：

小文是一位 10 歲的男孩，因為動靜脈畸形引起中風，造成次發性的半邊輕癱。他前來門診接受治療，他的父母親特別在意小文手腳無力的問題。您正在

考慮實施密集的特定任務肌力訓練計畫，但您的同事警告您，這樣的訓練可能會增加病人的中度屈肌張力與痙攣，建議執行低強度牽拉與被動擺位計畫。

**第 1 題：**請選擇上述其中一個臨床情境，為該情境寫出一個明確的臨床問題，以幫助您搜尋臨床文獻來回答病人的問題。

**第 2 題：**您可以從哪裡找到這個或其他類似臨床問題的答案？請盡量寫出所有可能的資訊來源(不限於那些您認為「好」的資料來源)，並說明各種您列出的資訊來源的優缺點。

**第 3 題：**哪一種類型的研究(研究設計)最能解答您在第 1 題提出的臨床問題？為什麼？

**第 4 題：**如果您要使用 Medline、CINAHL 或其它資料庫搜尋原著型研究，來回答您在第 1 題所提出的臨床問題，您會使用怎樣的搜尋策略？請盡可能具體描述您會使用的搜尋詞彙和搜尋欄位，並解釋這樣搜尋的原因。為了得到最適合的文章，請描述您會如何對搜尋做限制，並說明理由。

**第 5 題：**當您找到一篇有關該問題或任何其他問題的原著型研究報告時，您會考慮該研究的哪些特徵來決定它是否與您的問題相關？請舉例說明。第 6 題與第 7 題將詢問您如何決定研究的效度及結果的重要性。本題請著重在您如何決定研究內容與您的臨床實踐相關。

第 6 題：當您找到一篇有關您臨床問題或任何其它問題的原著型研究報告時，您會考慮該研究的哪些特徵來決定它的結果是否具有效度？請舉例說明。(第 7 題將詢問您如何決定研究結果的重要性。本題請著重在研究的效度。)

**第 7 題：**當您找到一篇有關您臨床問題或任何其它問題的原著型研究報告時，您會考慮該研究結果的哪些特徵來確定它們在臨床上的重要性與統計學上的顯著性？

第 8 題：請根據您選擇的臨床情境，列出最多兩個您會詢問病人或其家屬的問題，以更能了解他們的個人偏好，或與您臨床問題相關的情況。

第 9 題：有一篇關於跑步機運動心電圖測試(exercise treadmill testing，簡稱 ETT)在診斷冠狀動脈疾病(coronary artery disease，簡稱 CAD)的診斷準確性研究中，共有 120 位疑似患有 CAD 的女性，其中 30 位後來被判定患有 CAD(依據單一或多條冠狀動脈血管狹窄>50%)。在患有 CAD 的病患中，10 人的 ETT 結果為異常。在 90 位被判定沒有 CAD 的病患中，有 30 位 ETT 結果為異常。

根據以上結果，請問：

a. ETT 診斷 CAD 的敏感性(sensitivity)為________

b. ETT 診斷 CAD 的陽性預測值(positive predictive value)為________

c. ETT 診斷 CAD 的陽性相似比(positive likelihood ratio)為________

第 10 題：最近一篇針對患有尿失禁孕婦的隨機分配試驗發現，在分娩後 3 個月，有 20%的受試者在骨盆底肌訓練後出現尿失禁，而對照組有 30%。該研究的統計顯著水準設在 alpha 值為 0.05。

根據以上結果：

a. 尿失禁復發的絕對風險降低值(absolute risk reduction)為________

b. 尿失禁復發的相對風險降低值(relative risk reduction)為________

c. 尿失禁復發的益一需治數(number needed to treat)為________

d. 表示組間有統計上顯著差異的 p 值為________

第 11 題：第 10 題中的同一項研究顯示，接受骨盆底肌訓練的女性發生尿失禁的相對風險為 0.66，表示骨盆底肌訓練可以降低尿失禁的風險。當我們想知道這個結果是否有統計上的顯著性時，會檢視信賴區間。怎樣的信賴區間會支持實驗組與對照組間的尿失禁比例確實有統計上顯著差異的結論？

**第 12 題：**哪一種研究設計最適合用於診斷的研究？

**第 13 題：**哪一種研究設計最適合用於預後的研究？
